# A Comparative Analysis of Diabetic Ketoacidosis (DKA) in Sodium-Glucose Cotransporter-2 Inhibitor (SGLT2i) Users Versus Non-users: A Systematic Review

**DOI:** 10.7759/cureus.93456

**Published:** 2025-09-29

**Authors:** Sawsan Abdelwahab Abdella Altom, Ali Hadi M Alhajri, Hiba Karimeldin Mohamed Ali, Dalia Abdelrhman Mohammed Ahmed, Marwa AbdalGader Rabah Rahma, Tartel Abdelhamed Mohamed Ahmed, Marwa Mohamoud Mohmmed Abd Alatif, Rowida Abdeen Hassan Ahmed

**Affiliations:** 1 Clinical Biochemistry, The Royal Infirmary of Edinburgh, Edinburgh, GBR; 2 Endocrinology, Najran Armed Forces Hospital, Ministry of Defense Health Services, Najran, SAU; 3 Internal Medicine, Russells Hall Hospital, Dudley, GBR; 4 Internal Medicine, Al-Quwayiyah Primary Health Care, Al-Quwayiyah, SAU; 5 Internal Medicine, Dubai Medical University Hospital, Dubai, ARE; 6 Internal Medicine, Nizwa Hospital, Nizwa, OMN; 7 Internal Medicine, Shendi University, Shendi, SDN; 8 General Medicine, NMC Royal Hospital, Abu Dhabi, ARE

**Keywords:** clinical outcomes, diabetic ketoacidosis, euglycemic dka, incidence, risk factors, sglt2 inhibitors

## Abstract

Diabetic ketoacidosis (DKA) remains a life-threatening complication of diabetes mellitus, with emerging concerns about its association with sodium-glucose cotransporter-2 inhibitors (SGLT2i). While SGLT2i offer significant cardiovascular and renal benefits, their potential to increase DKA risk - particularly euglycemic diabetic ketoacidosis (eDKA) - warrants systematic evaluation.

This review aims to compare the incidence, clinical features, and outcomes of DKA in SGLT2i users versus non-users. A comprehensive literature search was conducted across PubMed/MEDLINE, Scopus, CINAHL, IEEE Xplore, and Web of Science. Eligible studies included comparative analyses of DKA in SGLT2i users and non-users, with retrospective and prospective designs. Data were extracted on incidence, laboratory features, precipitating factors, and clinical outcomes. Risk of bias was assessed using the Newcastle-Ottawa Scale. Thirteen studies were included, encompassing diverse populations and regions. SGLT2i users exhibited a higher incidence of DKA, with a notable proportion presenting as eDKA. Key distinguishing features included lower blood glucose levels, higher sodium concentrations, and prolonged hospital stays. Surgery and infections were common triggers, while mortality rates remained comparable between groups. Methodological quality was generally high, though heterogeneity precluded meta-analysis. SGLT2i use is associated with an increased risk of DKA, particularly euglycemic variants, necessitating heightened clinical vigilance. Temporary discontinuation during high-risk periods and early diagnostic suspicion are recommended. Standardized criteria for eDKA and further prospective studies are needed to optimize patient safety.

## Introduction and background

Diabetic ketoacidosis (DKA) is a serious complication of diabetes mellitus, classically linked to insulin deficiency and poor glycemic control [1,2]. Despite advances in treatment, it continues to cause significant morbidity, hospitalizations, and healthcare costs. Although historically more common in type 1 diabetes mellitus (T1DM), DKA is now increasingly observed in type 2 diabetes mellitus (T2DM), especially with the use of newer therapies [3].

Sodium-glucose cotransporter-2 inhibitors (SGLT2i) have become widely adopted for their benefits in glycemic control, weight reduction, and cardiovascular and renal outcomes [4]. However, they are also associated with DKA, including euglycemic diabetic ketoacidosis (eDKA), where glucose levels may not be markedly elevated, delaying diagnosis and treatment. Proposed mechanisms include enhanced ketogenesis, reduced insulin, and increased glucagon secretion [5].

Evidence comparing DKA in SGLT2i users versus non-users remains limited and inconsistent [6]. While some studies suggest an increased risk, absolute rates are low, and the clinical presentation often differs from traditional DKA. Furthermore, most reports focus on specific subgroups or agents, leaving uncertainty about the broader clinical implications.

Given the expanding use of SGLT2i across diverse diabetic populations, clarifying the incidence, clinical features, and outcomes of DKA in users versus non-users is critical. This systematic review aims to synthesize the available evidence, address current gaps, and guide safer therapeutic use of this drug class.

## Review

Methodology

Study Design and Protocol

This systematic review was conducted in accordance with the Preferred Reporting Items for Systematic Reviews and Meta-Analyses (PRISMA) 2020 guidelines. The review protocol was not prospectively registered on a platform such as PROSPERO due to significant time constraints.

Eligibility Criteria

The eligibility criteria were defined based on the PICOS framework (Population, Intervention, Comparator, Outcomes, Study Design) to ensure a structured and transparent selection process. Studies were included if they met the following criteria, as detailed in Table 1.

**Table 1:** PICOS Framework for Study Eligibility PICOS, Population, Intervention, Comparator, Outcomes, Study Design

Component	Eligibility Criteria
Population	Adult patients (≥18 years) with a diagnosis of type 1 or type 2 diabetes mellitus.
Intervention	Treatment with any sodium-glucose cotransporter-2 (SGLT2) inhibitor (e.g., canagliflozin, dapagliflozin, empagliflozin, ertugliflozin).
Comparator	Patients with diabetes not treated with SGLT2 inhibitors (e.g., on other antihyperglycemic agents or insulin).
Outcomes	Primary: Incidence of diabetic ketoacidosis (DKA). Secondary: Proportion of euglycemic DKA (eDKA), clinical features at presentation (e.g., blood glucose, pH, bicarbonate), precipitating factors, and clinical outcomes (e.g., length of hospital stay, mortality).
Study Design	Comparative studies, including randomized controlled trials (RCTs), prospective or retrospective cohort studies, and case-control studies.

Studies were excluded if they were case reports, case series, narrative reviews, systematic reviews, meta-analyses, conference abstracts without available full text, or non-English publications. This was to maintain methodological rigor and the comparability of findings across studies.

Information Sources

A comprehensive literature search was performed across multiple electronic databases from their inception until August 2025 to ensure wide coverage of all relevant studies published since the approval of SGLT2i. The databases searched included PubMed/MEDLINE, Scopus, CINAHL, IEEE Xplore, and Web of Science. These databases were selected to capture both clinical and interdisciplinary studies, including biomedical, nursing, and pharmacological research relevant to SGLT2i use and DKA outcomes. Additional sources, such as the reference lists of included articles, were also screened to identify studies not captured during the initial search.

Search Strategy

A structured search strategy was developed using a combination of controlled vocabulary (e.g., MeSH terms in PubMed) and free-text keywords tailored to each database (Table 6, see Appendix). The search concepts targeted three key elements: (1) SGLT2i, (2) DKA, and (3) diabetes mellitus. Boolean operators were applied to combine terms within each concept using "OR," and to combine the different concepts using "AND." The search was restricted to studies on human subjects. No language filters were applied initially. The detailed search strategy for each database is included in Table 2.

**Table 2:** Literature Search Results by Database (as of August 2025)

Database	Date of Search	Search Terms/Strategy	Number of Records Identified
Scopus	August 16, 2025	TITLE-ABS-KEY((sglt2 AND inhibitor*) OR "sodium-glucose cotransporter 2") AND TITLE-ABS-KEY(("diabetic ketoacidosis" OR dka OR "euglycemic dka")) AND TITLE-ABS-KEY(diabetes)	128
PubMed/MEDLINE	August 15, 2025	(SGLT2 inhibitor* OR sodium-glucose cotransporter 2 inhibitor*) AND (diabetic ketoacidosis OR DKA OR euglycemic DKA) AND (diabetes)	112
CINAHL	August 17, 2025	(TI (sglt2 inhibitor* OR "sodium-glucose cotransporter 2") OR AB (sglt2 inhibitor* OR "sodium-glucose cotransporter 2")) AND (TI ("diabetic ketoacidosis" OR dka OR "euglycemic dka") OR AB ("diabetic ketoacidosis" OR dka OR "euglycemic dka")) AND (TI diabetes OR AB diabetes)	34
IEEE Xplore	August 17, 2025	("SGLT2 inhibitors" OR "sodium-glucose cotransporter 2") AND ("diabetic ketoacidosis" OR DKA)	58
Web of Science	August 16, 2025	TS=((sglt2 inhibitor*) OR "sodium-glucose cotransporter 2") AND TS=("diabetic ketoacidosis" OR dka OR "euglycemic dka") AND TS=(diabetes)	63
Other Sources	August 2024	Citation searching of included articles and relevant reviews	14
Total Records			409

Selection Process

The selection of studies was conducted in two stages. First, all records retrieved from the database searches were imported into EndNote X9 (Clarivate, London, United Kingdom), where duplicate references were automatically identified and manually confirmed for removal. Following de-duplication, two reviewers independently screened the titles and abstracts of the remaining records to identify potentially relevant studies. In the second stage, full-text articles of shortlisted studies were assessed against the eligibility criteria. Any discrepancies between reviewers were resolved through discussion, and when necessary, consultation with a third reviewer was sought to reach consensus. The entire selection process was documented using a PRISMA 2020 flow diagram.

Data Collection Process

Data extraction was carried out independently by two reviewers using a standardized extraction form designed for this review. Extracted information included study characteristics (authors, year, country, study design, and sample size), participant characteristics (type of diabetes, age, and baseline clinical features), details of SGLT2i use (drug type, duration, and comparator), and DKA-related outcomes. Discrepancies in extracted data were discussed and resolved by consensus.

Data Items

The primary outcome of interest was the comparative incidence of DKA in SGLT2i users versus non-users. Secondary outcomes included the proportion of eDKA, mean time to onset of DKA, severity of presentation, and associated clinical outcomes such as length of hospital stay or mortality. Additional contextual data, such as patient comorbidities and concurrent medication use, were also extracted when available.

Study Risk of Bias Assessment

The methodological quality and risk of bias for the included observational studies were assessed using the Newcastle-Ottawa Scale (NOS) [7], which evaluates selection of study groups, comparability of groups, and outcome assessment. Two reviewers performed the risk of bias assessment independently, and disagreements were resolved through discussion. Studies were classified as low, moderate, or high risk of bias, based on NOS scores.

Effect Measures

Effect measures, such as risk ratios (RRs), odds ratios (ORs), or hazard ratios (HRs), as reported in the primary studies, were extracted and presented narratively. No new effect estimates were calculated in this review to preserve the validity of the original analyses.

Synthesis Methods

Given the expected heterogeneity in study populations, diagnostic criteria for DKA, study designs, and outcome definitions across the included studies, a quantitative meta-analysis was not conducted. Pooling of results would have risked generating misleading effect estimates due to methodological and clinical variability. Instead, a narrative synthesis was performed, summarizing findings across studies and highlighting consistencies, discrepancies, and emerging trends. Where feasible, tabular comparisons were provided to aid interpretation of the results.

Reporting Bias Assessment

To minimize the risk of reporting bias, all included studies were systematically assessed for selective outcome reporting by comparing reported outcomes with study objectives stated in the methods section of the publications. Discrepancies or suspected selective reporting were noted during data extraction.

Results

Studies Selection Process

**Figure 1:**
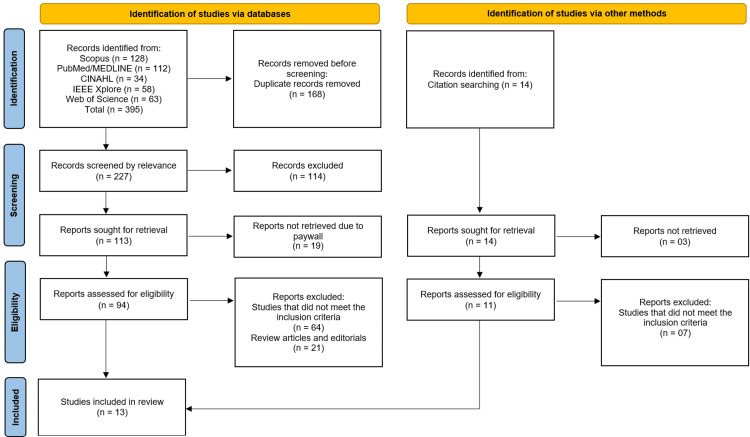
PRISMA Flowchart PRISMA, Preferred Reporting Items for Systematic Reviews and Meta-Analyses

The study selection process followed the PRISMA guidelines and involved multiple stages of screening. Initially, 395 records were identified through database searches (Scopus: n = 128, PubMed/MEDLINE: n = 112, CINAHL: n = 34, IEEE Xplore: n = 58, Web of Science: n = 63) and citation searching (n = 14). After removing 168 duplicate records, 227 studies underwent relevance screening, of which 114 were excluded. Subsequently, 113 full-text reports were sought for retrieval, with 19 unavailable due to paywalls, leaving 94 reports for eligibility assessment. Among these, 64 studies were excluded for not meeting the inclusion criteria, and 21 were removed as review articles or editorials. Additionally, 14 reports from citation searching were assessed, with seven excluded for irrelevance. Ultimately, 13 studies fulfilled the eligibility criteria and were included in the systematic review (Figure 1) [8-20].

Study Characteristics

The systematic review included 13 studies investigating DKA in patients using SGLT2i compared to non-users [8-20]. The studies were conducted across diverse regions, including the United States [8,9], Israel [10,14,16], China [11], Greece [12], Australia [13,18], Canada [15], Korea [17], Turkey [19], and the United Arab Emirates [20]. Study designs were predominantly retrospective, encompassing cohort studies [8,10,14,16,18], case-control studies [9,11,13,19], and retrospective analyses [12,15,17,20]. Sample sizes ranged from 18 to 34,671 [8,12], with most studies focusing on T2DM, while a few included mixed populations (T1DM and T2DM) [10,16,17,19]. Follow-up durations varied widely, from 14 days post-surgery to a median of 4.4 years [8,10]. Diagnostic criteria for DKA included clinical records, laboratory findings (e.g., pH <7.35, positive ketones), and adherence to international guidelines (Table 3).

**Table 3:** Characteristics of Included Studies SGLT2i, Sodium-Glucose Cotransporter 2 inhibitors; DM, Diabetes Mellitus; T1DM, Type 1 Diabetes Mellitus; T2DM, Type 2 Diabetes Mellitus; DKA, Diabetic Ketoacidosis; eKA, Euglycemic Ketoacidosis; euDKA, Euglycemic Diabetic Ketoacidosis; VA, Veterans Affairs; CABG, Coronary Artery Bypass Grafting; NR, Not Reported; HCO₃, Bicarbonate; CO₂, Carbon Dioxide; JBDS, Joint British Diabetes Societies; AACE, American Association of Clinical Endocrinology; ACE, American College of Endocrinology; UAE, United Arab Emirates

First Author (Year)	Country/Region	Study Design	Population (Type 1/Type 2 DM/Mixed)	Sample Size (n)	SGLT2i Drug(s) Used	Comparator Group	Mean Age (Years)	Follow-Up Duration	Diagnostic Criteria for DKA
Dixit et al. (2025) [8]	United States (Nationwide, commercial & Medicare insurance data)	Retrospective cohort study	T2DM	34,671 (SGLT2i users: 2,607; Non-users: 32,064)	SGLT2i	Non-users of SGLT2i	63.9 ± 14.0	14 days post-surgery	Diagnosis codes for DKA
Tallarico et al. (2025) [9]	United States (VA Health Care System, multicenter)	Retrospective, propensity-matched case-control study	T2DM (predominantly, as SGLT2i not approved for T1DM in the VA setting)	7,448 SGLT2i users vs. 455,520 non-users (matched: 7,439 vs. 33,489)	SGLT2i	Non-users (1:5 propensity-matched controls)	Users: 67.7 (±8.1); Non-users: 65.8 (±11.0)	30 days post-surgery	Postoperative eKA
Tsur et al. (2025) [10]	Israel	Retrospective cohort	Insulin-deficient phenotype (Mixed DM)	12,954 (6,572 SGLT2i users; 6,382 non-users)	SGLT2i	Non-users (matched cohort)	18-70 years (mean not specified)	Median 4.4 years	First DKA event (clinical diagnosis, prior history excluded)
Hu et al. (2025) [11]	China (Tianjin)	Single-centre, nested case-control study (CABG patients)	T2DM	1,524 (20 euDKA cases: 13 SGLT2i users, 7 non-users)	SGLT2i	Non-users (95 controls in nested case-control)	64 (±9)	6 months (postoperative follow-up)	Laboratory findings: arterial blood gas (pH <7.35), urine ketones, blood glucose <10 mmol/L
Papanastasiou et al. (2021) [12]	Greece	Retrospective study	T2DM, as SGLT2i are mostly used in T2DM	18 (6 SGLT2i users with DKA, 12 non-users with DKA)	SGLT2i	Patients with DKA due to other causes	NR	2018-2020 (retrospective review period)	Based on clinical and biochemical characteristics (pH, HCO3, CO2, anion gap)
O’Brolchain et al. (2024) [13]	Australia (Queensland, two teaching hospitals)	Retrospective observational study	T2DM (Adults >18 years)	165 (94 SGLT2i users, 70 non-users)	SGLT2i	Non-SGLT2i users	NR	April 2015-January 2022	Joint British Diabetes Society (JBDS) and American Association of Clinical Endocrinology/American College of Endocrinology (AACE/ACE) criteria
Nakhleh et al. (2023) [14]	Israel (Rambam Health Care Campus)	Retrospective analysis	T2DM	71 (16 SGLT2i users; 55 non-users)	SGLT2i	T2DM patients with DKA not on SGLT2i	NR	Median follow-up: 35.1 months (users), 36.7 months (non-users)	Clinical + laboratory confirmation from records
Khan et al. (2024) [15]	Canada (Montfort Hospital, Ottawa)	Retrospective Case Series	T2DM	88 (33 SGLT2i users, 55 non-users)	SGLT2i	Non-SGLT2i users	NR	NR	Patients admitted with DKA (clinical diagnosis from medical records)
Keler et al. (2024) [16]	Israel (Shamir Medical Center)	Retrospective cohort study	Mixed (T1DM and T2DM)	204	SGLT2i	Non-SGLT2i users (within both T1DM and T2DM groups)	T2DM patients described as older than T1DM	2013-2021 (hospital admissions period, not individual follow-up)	Standard clinical diagnosis of DKA implied
Jeon et al. (2019) [17]	Korea (9 centers)	Retrospective multicenter study	Mixed (majority T2DM, 51%)	523 DKA episodes (15 SGLT2i users)	SGLT2i	Non-users of SGLT2i	NR	Sept 2014-Apr 2017 (≈ 2.5 years)	DKA diagnosis based on admission records
Hamblin et al. (2019) [18]	Australia (Melbourne & Geelong)	Retrospective, multicenter, controlled cohort study	T2DM	162 (37 SGLT2i users, 125 non-users)	SGLT2i	Non-SGLT2i users with T2DM	NR	Sept 1, 2015-Oct 31, 2017 (~2 years)	Physician-adjudicated diagnosis of DKA (with recognition of euglycemic cases: glucose <250 mg/dL in some users)
Genc et al. (2024) [19]	Turkey	Comparative observational (case-control style)	Mixed (DM patients with DKA)	51 (19 SGLT2i users, 32 non-users)	SGLT2i	DKA patients not treated with SGLT2i	SGLT2i users significantly older than non-users	NR	Diagnosis of DKA
Almazrouei et al. (2023) [20]	UAE (Tawam Hospital, Al Ain City)	Retrospective study	T2DM	55	SGLT2i	Non-SGLT2i users	54.0 ± 18.9	Jan 2017-Mar 2021 (~4 years, retrospective)	Based on hospital admission records

Incidence of DKA

The incidence of DKA was consistently higher among SGLT2i users compared to non-users, though the magnitude varied across studies. Dixit et al. [8] reported that 31% of DKA cases occurred in SGLT2i users, while Almazrouei et al. [20] observed a similar proportion (31%). In contrast, Tsur et al. [10] found a modest increase in DKA incidence among SGLT2i users (2.22% vs. 1.54% in non-users). Notably, Hu et al. [11] identified a 7.6% incidence of DKA in SGLT2i users who underwent coronary artery bypass grafting (CABG), compared to 0.15% in non-users. eDKA, characterized by near-normal blood glucose levels, was disproportionately reported in SGLT2i users. For instance, 56.3% of DKA cases in SGLT2i users were euglycemic, compared to only 2.6% in non-users [8-20]. Similarly, Hamblin et al. [18] observed eDKA in 41% of SGLT2i users versus 0.8% of non-users (Table 4).

**Table 4:** Comparative Outcomes of DKA in SGLT2i Users vs. Non-users DKA, Diabetic Ketoacidosis; SGLT2i, Sodium-Glucose Cotransporter 2 inhibitors; eKA, Euglycemic Ketoacidosis; euDKA, Euglycemic Diabetic Ketoacidosis; SBP, Systolic Blood Pressure; Na, Sodium; OR, Odds Ratio; NR, Not Reported; HbA1c, Hemoglobin A1c; BMI, Body Mass Index; CABG, Coronary Artery Bypass Grafting; HCO₃⁻, Bicarbonate; JBDS, Joint British Diabetes Societies; AACE, American Association of Clinical Endocrinology; ACE, American College of Endocrinology; CI, Confidence Interval

First Author (Year)	Incidence of DKA (%) - Users	Incidence of DKA (%) - Non-users	Euglycemic DKA Cases (%)	Laboratory Findings (Glucose, pH, HCO₃⁻, Ketones)	Precipitating Factors (Surgery, Infection, Fasting, Insulin Omission)	Mortality (%)	Length of Hospital Stay (Days)
Dixit et al. (2025) [8]	17/55 (31%) of DKA cases occurred in SGLT2i users	38/55 (69%) of DKA cases occurred in non-users	56.3% in SGLT2i users vs. 2.6% in non-users	Lower glucose in users (16.2 mmol/L vs. 24.9 mmol/L in non-users); Lower SBP in users (119.9 vs. 140 mmHg); Higher Na in users (137.5 vs. 132.6 mmol/L)	Infection most common (47% of users, 8/17); Other factors not detailed	No significant difference between groups	Users had ~5× higher risk of prolonged stay ≥14 days; mean duration not reported
Tallarico et al. (2025) [9]	Increased risk (OR 1.11)	Lower than users (reference group)	Postoperative eKA	NR	Surgery (postoperative trigger)	1.1% (users) vs. 1.6% (non-users)	Median 6 days with eKA vs. 3 days without eKA
Tsur et al. (2025) [10]	2.22% (143/6,572)	1.54% (96/6,382)	NR	NR	HbA1c >9%, BMI ≤25, Insulin use	NR	NR
Hu et al. (2025) [11]	13/171 (7.6% when SGLT2i stopped 1-6 days before CABG); 1% overall incidence in cohort	2/1,353 (~0.15%)	100% (all 15 identified DKA cases were euglycemic)	pH <7.35; Blood glucose <10 mmol/L; Ketones positive	Major surgery (CABG); timing of last SGLT2i dose (≤3 days pre-op increased risk)	NR	NR
Papanastasiou et al. (2021) [12]	6 cases	12 cases	Higher likelihood (median glucose 229 mg/dL, some <250 mg/dL)	Users: Glucose 229 (150-481) mg/dL; Non-users: 458.5 (332-695) mg/dL; pH & HCO₃⁻: no significant difference	NR	NR	Users: 11 (6-22); Non-users: 5.5 (2-14)
O’Brolchain et al. (2024) [13]	94 patients (diagnosed DKA, proportion meeting criteria: 56% JBDS, 63% AACE/ACE)	70 patients (diagnosed DKA, proportion meeting criteria: 72% JBDS, 82% AACE/ACE)	NR	NR	NR	NR	NR
Nakhleh et al. (2023) [14]	16/71 (22.5%) of DKA cases were SGLT2i users	55/71 (77.5%) of DKA cases were non-users	NR	NR	Infections mentioned (rates comparable between groups)	In-hospital: comparable; Long-term: 12.5% (users) vs. 52.7% (non-users), p = 0.004	NR
Khan et al. (2024) [15]	33/88 (37.5%)	55/88 (62.5%)	NR	NR	NR	NR	NR
Keler et al. (2024) [16]	NR (SGLT2i group mentioned, no incidence reported)	NR	EuDKA linked with SGLT2i	NR	Triggers mentioned: SGLT2i, but no breakdown	Higher in T2DM: 6.4% in-hospital, 7.7% at 90 days; SGLT2i use did not change mortality	Prolonged hospital stay
Jeon et al. (2019) [17]	Rare (15/523 cases, ~2.9%)	Majority (~97.1%)	NR	Glucose: 413 mg/dL (users) vs. 554 mg/dL (non-users)	NR	0% (users) vs. 3% overall	11 days (average, both groups)
Hamblin et al. (2019) [18]	1.02 per 1000 (95% CI: 0.74-1.41)	0.69 per 1000 (95% CI: 0.58-0.82)	41% (15/37) vs. 0.8% (1/125)	Peak glucose <250 mg/dL in 41% of SGLT2i users vs. 0.8% non-users	NR	NR	NR
Genc et al. (2024) [19]	19/51 (37.3%) (users with DKA in sample)	32/51 (62.7%) (non-users with DKA in sample)	Present only in users (p = 0.005)	Glucose lower in users vs. higher in non-users (p = 0.006); Chloride higher in users (p = 0.036); pH, HCO₃⁻, ketones NR	Genitourinary infections (urinary tract, vulvovaginitis) more common in users (p-values: 0.036, 0.001, 0.005, 0.003)	NR	NR
Almazrouei et al. (2023) [20]	17/55 (31%) of DKA cases occurred in SGLT2i users	38/55 (69%) of DKA cases occurred in non-users	56.3% in SGLT2i users vs. 2.6% in non-users	Lower glucose in users (16.2 mmol/L vs. 24.9 mmol/L in non-users); Lower SBP in users (119.9 vs. 140 mmHg); Higher Na in users (137.5 vs. 132.6 mmol/L)	Infection most common (47% of users, 8/17); Other factors not detailed	No significant difference between groups	Users had ~5× higher risk of prolonged stay ≥14 days; mean duration not reported

Laboratory and Clinical Features

DKA in SGLT2i users exhibited distinct laboratory profiles, including lower blood glucose levels and higher sodium concentrations. Dixit et al. [8] reported mean glucose levels of 16.2 mmol/L in users versus 24.9 mmol/L in non-users, alongside lower systolic blood pressure (119.9 vs. 140 mmHg). Papanastasiou et al. [12] noted median glucose levels of 229 mg/dL in users compared to 458.5 mg/dL in non-users, though pH and bicarbonate levels did not differ significantly. Genc et al. [19] corroborated these findings, with users demonstrating lower glucose and higher chloride levels (p = 0.006 and p = 0.036, respectively). These trends underscore the unique metabolic perturbations associated with SGLT2i-induced DKA.

Precipitating Factors

Surgery and infections emerged as prominent triggers for DKA in SGLT2i users. Hu et al. [11] identified major surgery (CABG) as a key precipitant, particularly when SGLT2i were discontinued ≤3 days preoperatively. Infections, especially genitourinary, were more common in users (47% of cases) [8,19]. Tallarico et al. [9] highlighted postoperative eDKA as a specific risk, with an OR of 1.11 for SGLT2i users. Other factors, such as insulin omission or fasting, were less frequently reported, but contributed to DKA development in both groups.

Clinical Outcomes

Mortality rates were generally comparable between SGLT2i users and non-users, though some studies noted divergent long-term outcomes. Nakhleh et al. [14] reported no significant in-hospital mortality difference but observed higher long-term mortality in non-users (52.7% vs. 12.5%, p = 0.004). Hospital stays were prolonged in SGLT2i users, with Dixit et al. [8] noting a fivefold higher risk of stays ≥14 days. Keler et al. [16] also described extended hospitalization for SGLT2i-associated DKA, though mortality remained unaffected by SGLT2i use. Adherence to diagnostic criteria for DKA was lower in SGLT2i users (56%, Joint British Diabetes Societies (JBDS) criteria) compared to non-users (72%) [13], potentially reflecting diagnostic challenges posed by eDKA.

Risk of Bias Findings

The risk of bias assessment using the NOS revealed that most included studies demonstrated low-to-moderate risk, with scores ranging from 4 to 9 out of 9. Low-risk studies (score ≥7) [8,9,11,13,14,16-20] were characterized by robust selection criteria, adjustments for confounders, and reliable outcome assessment. Moderate-risk studies (score 4-6) included Tsur et al. [10] (6/9), Papanastasiou et al. [12] (5/9), and Khan et al. [15] (4/9), primarily due to limitations in comparability (e.g., unadjusted confounders) or smaller sample sizes. Notably, Tallarico et al. [9] and Hamblin et al. [18] achieved perfect scores (9/9) owing to propensity matching and controlled designs, while Khan et al. [15] scored lowest (4/9) due to their retrospective case-series design and lack of a control group. Overall, the findings support the methodological rigor of the majority of studies, though caution is warranted when interpreting results from moderate-risk studies (Table 5).

**Table 5:** Risk of Bias Results on Newcastle-Ottawa Scale (NOS) Tool

First Author (Year)	Study Design	Selection (Max 4)	Comparability (Max 2)	Outcome/Exposure (Max 3)	Total Score (Max 9)	Risk of Bias
Dixit et al. (2025) [8]	Retrospective cohort	4	2	2	8	Low
Tallarico et al. (2025) [9]	Propensity-matched case-control	4	2	3	9	Low
Tsur et al. (2025) [10]	Retrospective cohort	3	1	2	6	Moderate
Hu et al. (2025) [11]	Nested case-control	3	2	2	7	Low
Papanastasiou et al. (2021) [12]	Retrospective study	2	1	2	5	Moderate
O'Brolchain et al. (2024) [13]	Retrospective observational	3	1	3	7	Low
Nakhleh et al. (2023) [14]	Retrospective analysis	3	2	2	7	Low
Khan et al. (2024) [15]	Retrospective case series	2	1	1	4	Moderate
Keler et al. (2024) [16]	Retrospective cohort	3	1	3	7	Low
Jeon et al. (2019) [17]	Retrospective multicenter	3	1	3	7	Low
Hamblin et al. (2019) [18]	Controlled cohort study	4	2	3	9	Low
Genc et al. (2024) [19]	Case-control	3	2	2	7	Low
Almazrouei et al. (2023) [20]	Retrospective study	3	2	2	7	Low

Discussion

The findings of this systematic review provide a comprehensive synthesis of the comparative risks, clinical features, and outcomes of DKA in patients using SGLT2i versus non-users. The analysis of 13 studies [8-20], encompassing diverse geographical regions and study designs, reveals several critical insights into the association between SGLT2i use and DKA, particularly highlighting the unique challenges posed by eDKA. The incidence of DKA was consistently higher among SGLT2i users, though the magnitude of this risk varied across studies. For instance, Dixit et al. [8] and Almazrouei et al. [20] reported that 31% of DKA cases occurred in SGLT2i users, while Tsur et al. [10] observed a more modest increase (2.22% vs. 1.54% in non-users). This variability may reflect differences in study populations, follow-up durations, and diagnostic criteria. Notably, the predominance of eDKA among SGLT2i users - reported in 56.3% of cases by Dixit et al. [8] and 41% by Hamblin et al. [18] - underscores a distinct pathophysiological mechanism. Unlike classical DKA, eDKA presents with near-normal blood glucose levels, often delaying diagnosis and complicating clinical management. This aligns with existing literature, emphasizing the role of SGLT2i in promoting ketogenesis through increased glucagon secretion and reduced insulin-mediated suppression of lipolysis, even in the absence of hyperglycemia [21,22].

The laboratory and clinical features of DKA in SGLT2i users further distinguish this population from non-users. Across multiple studies, SGLT2i-associated DKA was characterized by significantly lower blood glucose levels (e.g., 16.2 mmol/L vs. 24.9 mmol/L in non-users [8]) and metabolic derangements, such as higher sodium and chloride concentrations [19]. These findings corroborate earlier reports that SGLT2i-induced DKA often presents with milder hyperglycemia but pronounced ketosis, reflecting a shift in fuel metabolism toward fat utilization [23]. The lower systolic blood pressure observed in SGLT2i users during DKA episodes may also reflect the diuretic effects of these drugs [8], which can exacerbate volume depletion - a known precipitant of DKA. Such nuances highlight the importance of recognizing atypical presentations of DKA in SGLT2i users, as reliance on traditional diagnostic criteria (e.g., blood glucose >250 mg/dL) may lead to underdiagnosis. This is particularly relevant in perioperative settings, where Hu et al. [11] identified a 7.6% incidence of DKA in SGLT2i users undergoing CABG, compared to 0.15% in non-users. The timing of SGLT2i discontinuation before surgery emerged as a critical factor, with doses administered within three days preoperatively significantly increasing risk. These observations are consistent with guidelines recommending temporary cessation of SGLT2i prior to major surgery, though adherence remains inconsistent in clinical practice [24].

Precipitating factors for DKA in SGLT2i users were predominantly surgery and infections, particularly genitourinary infections [8,19]. The latter aligns with the pharmacological action of SGLT2i, which increases glucosuria and may predispose to urinary tract infections [25]. Tallarico et al. [9] further identified postoperative eDKA as a specific hazard, with an OR of 1.11 among SGLT2i users. This risk is likely multifactorial, involving preoperative fasting, surgical stress, and fluid shifts - all of which can exacerbate ketosis in the context of SGLT2i use. Interestingly, insulin omission, a classic trigger for DKA, was less frequently reported in SGLT2i users, suggesting that the mechanisms underlying SGLT2i-associated DKA may differ from those in non-users. This distinction has important implications for patient education and monitoring, as individuals on SGLT2i may develop DKA even with adherence to insulin therapy, particularly during intercurrent illness or physiological stress.

Clinical outcomes of DKA in SGLT2i users were marked by prolonged hospital stays, as noted by Dixit et al. [8], who reported a fivefold higher risk of stays ≥14 days. This may reflect the diagnostic challenges and delayed recognition of eDKA, as well as the need for cautious metabolic correction to avoid complications such as cerebral edema. However, mortality rates were generally comparable between SGLT2i users and non-users, with some studies noting divergent long-term outcomes. For example, Nakhleh et al. [14] observed higher long-term mortality in non-users (52.7% vs. 12.5%, p = 0.004), possibly due to differences in baseline comorbidities or glycemic control. The lower adherence to diagnostic criteria for DKA in SGLT2i users (56% vs. 72% for JBDS criteria [13]) further underscores the need for standardized diagnostic approaches that account for eDKA. Current criteria, which often emphasize hyperglycemia, may fail to capture SGLT2i-associated cases, leading to underreporting and mismanagement.

The risk of bias assessment using the NOS revealed that most studies (10/13) were of low risk, with robust methodologies and adjustments for confounders. Propensity-matched designs, as employed by Tallarico et al. [9] and Hamblin et al. [18], were particularly effective in minimizing selection bias. However, moderate-risk studies, such as Papanastasiou et al. [12] and Khan et al. [15], were limited by small sample sizes and unadjusted confounders, warranting cautious interpretation of their findings. The overall methodological rigor supports the validity of the review’s conclusions, though heterogeneity in study designs and populations precluded meta-analysis.

Comparisons with existing literature reveal both consistencies and gaps. The elevated risk of eDKA with SGLT2i use has been well-documented in randomized trials and post-marketing surveillance [26], but real-world studies like those included here provide granular insights into precipitating factors and outcomes. For instance, the association between SGLT2i and postoperative DKA corroborates smaller case series [27], while the predominance of genitourinary infections as a trigger aligns with the drug’s known side effects [28]. However, the lower mortality observed in SGLT2i users contrasts with some prior reports [29], suggesting that the metabolic profile of eDKA may confer a survival advantage, or reflect differences in population characteristics.

This review has several limitations. First, the predominance of retrospective studies introduces potential biases, including unmeasured confounding and incomplete outcome data. Second, heterogeneity in diagnostic criteria for DKA and eDKA across studies complicates direct comparisons. Third, the lack of randomized controlled trials (RCTs) limits causal inference, though RCTs are ethically challenging for studying DKA. Finally, publication bias may favor studies reporting significant associations, though the inclusion of negative findings (e.g., comparable mortality) mitigates this concern.

## Conclusions

This review highlights the increased risk of DKA, particularly euglycemic variants, among SGLT2i users, driven by distinct metabolic perturbations and precipitating factors like surgery and infections. While mortality rates were similar, prolonged hospital stays and diagnostic challenges underscore the need for heightened vigilance in this population. Clinicians should consider temporary discontinuation of SGLT2i during high-risk periods and adopt a low threshold for evaluating DKA in symptomatic patients, even in the absence of hyperglycemia. Future research should focus on standardized diagnostic criteria for eDKA and prospective monitoring of SGLT2i-associated complications.

## Appendices

**Table 6:** Search Strategy

Database	Search String
PubMed/MEDLINE	(Diabetic Ketoacidosis[Mesh] OR "diabetic ketoacidosis"[tiab] OR "DKA"[tiab] OR "euglycemic diabetic ketoacidosis"[tiab] OR "EDKA"[tiab]) AND (Sodium-Glucose Transporter 2 Inhibitors[Mesh] OR "SGLT2 inhibitor*"[tiab] OR "sodium-glucose cotransporter 2 inhibitor*"[tiab] OR "dapagliflozin"[tiab] OR "empagliflozin"[tiab] OR "canagliflozin"[tiab] OR "ertugliflozin"[tiab])
Scopus	(TITLE-ABS-KEY("diabetic ketoacidosis" OR "DKA" OR "euglycemic diabetic ketoacidosis" OR "EDKA")) AND (TITLE-ABS-KEY("SGLT2 inhibitor*" OR "sodium-glucose cotransporter 2 inhibitor*" OR "dapagliflozin" OR "empagliflozin" OR "canagliflozin" OR "ertugliflozin"))
CINAHL (via EBSCOhost)	(MH "Diabetic Ketoacidosis" OR "diabetic ketoacidosis" OR "DKA" OR "euglycemic diabetic ketoacidosis" OR "EDKA") AND (MH "Sodium-Glucose Transporter 2 Inhibitors" OR "SGLT2 inhibitor*" OR "sodium-glucose cotransporter 2 inhibitor*" OR "dapagliflozin" OR "empagliflozin" OR "canagliflozin" OR "ertugliflozin")
IEEE Xplore	("diabetic ketoacidosis" OR "DKA" OR "euglycemic diabetic ketoacidosis" OR "EDKA") AND ("SGLT2 inhibitor*" OR "sodium-glucose cotransporter 2 inhibitor*" OR "dapagliflozin" OR "empagliflozin" OR "canagliflozin" OR "ertugliflozin")
Web of Science	TS=("diabetic ketoacidosis" OR "DKA" OR "euglycemic diabetic ketoacidosis" OR "EDKA") AND TS=("SGLT2 inhibitor*" OR "sodium-glucose cotransporter 2 inhibitor*" OR "dapagliflozin" OR "empagliflozin" OR "canagliflozin" OR "ertugliflozin")
